# Entrepreneurial intention and the three stages of entrepreneurial action: a process approach

**DOI:** 10.3389/fpsyg.2023.1184390

**Published:** 2023-07-21

**Authors:** Mzwakhe Dlamini, Melodi Botha

**Affiliations:** Department of Business Management, University of Pretoria, Pretoria, South Africa

**Keywords:** entrepreneurial intention, entrepreneurial action, entrepreneurial opportunity discovery stage, entrepreneurial opportunity evaluation stage, entrepreneurial opportunity exploitation stage

## Abstract

The relationship between entrepreneurial intention (EI) and entrepreneurial action (EA) is a popular topic in entrepreneurship research, owing to the contribution of these constructs in the process leading to the entrepreneurial activity taking place. There are still countries that are recording high entrepreneurial intention levels in comparison to their corresponding entrepreneurial action levels that are low. This is a global concern to which South Africa (SA) is also not immune. Most of the research tests the relationship between two single constructs: EI and EA. Our study follows a process approach and investigates the effect of this relationship between EI and the three stages of EA. A quantitative method was employed and a survey utilized whereby data was collected among 597 entrepreneurs in South Africa. The data was analyzed through Confirmatory Factor Analysis (CFA) and Structural Equation Modeling (SEM). The EI construct is supported through the Theory of Planned Behavior, in conjunction with the Motivation Opportunity Ability theory. The Discovery Theory, together with the Creative Theory, supports each of the stages of EA, namely: entrepreneurial opportunity discovery (EODI); entrepreneurial opportunity evaluation (EOEV); and entrepreneurial opportunity exploitation (EOEX). Previous research regarding the relationship between EI and EA measured this relationship from a binary point of view. This study contributes to the entrepreneurship field by employing the process approach to determine the impact of EI on the stages of EA. This study reveals that EI is statistically significant in all three stages of EA. However, the strength of this relationship is found to be strong between EI and the EODI and EOEV stages and moderate between EI and the EOEX stage. Therefore, this study reveals that effective training interventions and development are necessary between EI and the EOEX stage of EA.

## 1. Introduction

Most people desire to start their own businesses, yet their intentions do not always translate into action. This lack of action despite high levels of intention is a source of concern worldwide. South Africa (SA) is no different and despite being the second largest economy in Africa, there is still a low rate of individuals with the intention to start a business that actually go over into action (Bowmaker-Falconer and Meyer, 2022). Understanding the effect of this relationship has the potential to address the “*entrepreneurial intention-action”* (EI-EA) gap (Oliveira and Lima-Rua, 2018, p. 508; Van Gelderen et al., 2018, p. 924). This is as a result of the existence of a lack of exploiting entrepreneurial opportunities despite entrepreneurial intention (EI) being in place (Wiklund et al., 2017a, p. 3; Asante and Affum-Osei, 2019, p. 227).

As EI is defined as the state of mind that precedes EA (Esfandiar et al., 2019, p. 173), that is informed by either internal or external stimulus (Eid et al., 2019, p. 234). Building on the Theory of Planned Behavior (TPB; Ajzen, 1991) in conjunction with the Motivational Opportunity Ability (MOA) theory (Hui-Chen et al., 2014) has aided in formulating elements that suggested EI as the independent variable for this study. EA refers to theoretical and observable actions by an entrepreneur leading to entrepreneurial activities such as starting a business venture (Botha and Pietersen, 2020, p. 529). EA consists of distinct behavioral actions or, in this study, refers to the stages: Entrepreneurial Opportunity Discovery (EODI), Entrepreneurial Opportunity Evaluation (EOEV), and Entrepreneurial Opportunity Exploitation (EOEX; Shane and Venkataraman, 2000, p. 218; McMullen and Shepherd, 2006, p. 134; McMullen et al., 2007, p. 273). The elements of the Discovery Theory (DT), together with those of the Creative Theory (CT) were employed to formulate stages of EA (Shane and Venkataraman, 2000, p. 218; McMullen and Shepherd, 2006, p. 134).

The study followed the process approach method to determine the effect of EI into all three stages of EA. As EODI is a deliberate act of searching or identifying possible services or solutions that can be converted for profit (Hsieh et al., 2007, p. 1,255) and EOEV refers to an exercise to assess the feasibility and/or desirability of the entrepreneurial opportunities (Krueger, 1993, p. 5), it is crucial that EI forms the foundation of these stages. However, EOEX is a process by an entrepreneur leading to the gathering or recombining of required resources necessary to pursue the entrepreneurial opportunity (Ren et al., 2016, p. 468), so we assume that EI is not necessary here and that EA takes over.

However, the data was collected from 597 entrepreneurs consisting of nascent and existing entrepreneurs in SA, and we found that EI is statistically significant in all three stages of EA. The strength of EI is found to be strong between EI and EODI and EOEV stages, and moderate between EI and the EOEX stage.

Even though most research on the relationship between EI and EA investigates this relationship from a binary point of view (Bogatyreva et al., 2019, p. 309), the contribution of this study lies in adopting a process approach in determining the effect of EI to each of the stages of EA. Three contributions are found whereby this approach broadens our understanding of the impact of EI to each of the stages of EA. In particular is the fact that action is not only limited to when the product or the opportunity is fully exploited, but also to other entrepreneurial activities that are performed during the process leading to when the entrepreneurial opportunity is exploited. The contribution to theory lies in the four theories that support the relationship between EI and the three stages of EA. This study then shows that EI is necessary throughout the stages of EA in order for EA to be realized and possible the EI–EA gap to close, and that EI is not only necessary during the first stages of EA, namely opportunity discovery and evaluation, but also during the opportunity exploitation stage. This study contributes to a practice in which education and training programs can adopt to measure EA levels correctly, as opposed to the binary approach.

The study commences by presenting the background to the study, the literature review that aided in determining the hypotheses, then expounds on the research methodology followed, findings, contribution, limitations and concludes by offering recommendations for future research.

## 2. Background

Previous work on the relationship between EI and EA have contributed to the field of entrepreneurship (Bird and Schjoedt, 2017, p. 1; Esfandiar et al., 2019). Yet, the non-significant correlation between these constructs is intriguing (Meoli et al., 2020). Early research that investigated this relationship, cited action as being the direct result of intentions which was based on early theories that suggest intentions as a predictor for action (Ajzen, 1991, p. 179). However subsequent research found no significant correlation between intentions and action (Oliveira and Lima-Rua, 2018, p. 38), to suggest EA to be as a result of EI (Kautonen et al., 2015, p. 4). This prompted studies conducted by Wiklund et al. (2017a,b), Esfandiar et al. (2019), and Meoli et al. (2020) that investigated this relationship from the understanding of why EA levels are low despite their corresponding EI that is high.

Similarly in SA, the 2022 Global Entrepreneurship Monitor (GEM) report reveals the country as the second largest economy in Africa (by GDP) and with a relatively well-established markets and supply chains. However, 59.2% of the Total Entrepreneurial Activity (TEA) respondents found it difficult to translate EI into EA (Bowmaker-Falconer and Meyer, 2022). Meoli et al. (2020) are of the view that EA levels could be higher than what is currently recorded and the issue could be that they are not measured or communicated correctly in entrepreneurship. Further, scholars found that EA is not a unitary construct but consists of phases or stages (Shane and Venkataraman, 2000, p. 218; McMullen and Shepherd, 2006, p. 134; McMullen et al., 2007, p. 273). Therefore, we suggest that EA should not be investigated from a binary point but rather as a process approach by determining the effect of EI on the three stages of EA. In SA, limited studies have been conducted to measure the effect of EI on EA from a process approach.

## 3. Theoretical foundation and hypothesis development

EI and EA are well researched constructs in entrepreneurship due to their contribution leading to business venture taking place (Bird and Schjoedt, 2017, p. 1). Prior research on the relationship between intentions and action mainly focused on the formation and effects of the intentions construct and with limited research on EA (Adam and Fayolle, 2015, pp. 36–37; Esfandiar et al., 2019). The assumption being that intentions automatically lead to action (Van Gelderen et al., 2015, p. 658). Subsequent research in this regard found the impact of intentions on actions only accounts for a small percentage (Oliveira and Lima Rua, 2018, p. 508). However, emerging studies such as Meoli et al. (2020, p. 3) suggest that the effect of EI to EA could be higher than that stated in literature.

Most research on the relationship between EI and EA investigated this relationship from a binary point of view (Bogatyreva et al., 2019), even though EA is can be argued to be process driven than a once of event.

### 3.1. Entrepreneurial intention

**Figure 1:**
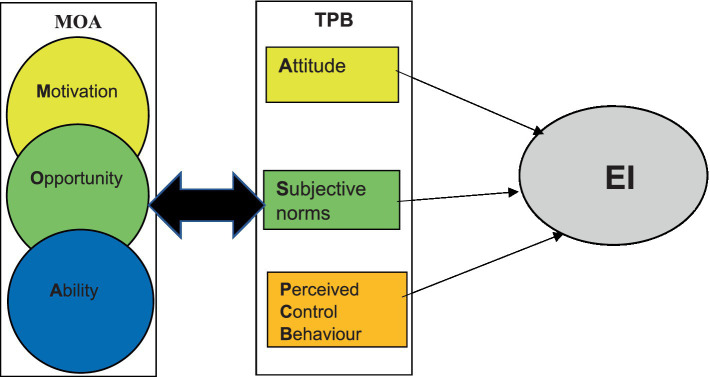
Entrepreneurial Intention model: adapted from Hui-Chen et al. (2014).

Entrepreneurship literature regard EI as key to play a vital role to enhance EA (Khan et al., 2022). EI is not born out of a vacuum but a result of influences driven by an internal or external stimulus (Eid et al., 2019). Previous research introduced factors and models that inform the formation of EI; the popular Shapero’s model of the entrepreneurial event (SEE) (Shapero and Sokol, 1982, p. 72) suggests intentions are as the result of external and internal stimuli. The recent study by Liu et al. (2022) integrated TPB and person-environment fit theory, and posited how perceived university support relates to students’ attitudes toward entrepreneurship, subjective norms, and entrepreneurial self-efficacy, which in turn impact EI. This study adopted Hui-Chen et al. (2014) model that integrate elements of the Theory of Planned Behavior (TPB) together with Motivation Opportunity Ability (MOA) theory as illustrated in Figure 1.

Figure 1 depicts the entrepreneurial intention model. That comprises of both elements of TPB and of MOA theories. By integrating MOA elements with those of TPB is likely to create intentions to stimulate action (Hui-Chen et al., 2014).

TPB over the years is regarded as the most proximal prognosticator of intentions to behavior (Richards et al., 2019). This theory is made up of three key elements as presented on Table 1.

**Table 1:** Elements of TPB.

Attitude	Subjective norms	Perceived control behavior
1. Attitude toward entrepreneurship	1. Imitating	1. Capabilities
2. Desire to be entrepreneur	2. External stimulus	2. Skills
3. Evaluation of opportunities	3. Succession plans	3. Knowledge
4. Willingness/Interest	4. Social desirability	4. Potential
Ajzen (1991), Whiteside and Lynam (2001), Esfandiar et al. (2019), Jumani and Sukhabot (2021), Suna et al. (2020)	Ajzen (1991), Malebana (2014), Bozer et al. (2017), Eid et al. (2019), Randerson et al. (2020)	Hui-Chen et al. (2014), Malebana (2014), Richards et al. (2019)

Table 1 presents elements that informs TPB and the literature that supports them namely; attitude, social norms and perceived control behavior. This suggests that in order for the intention to be realizable is likely to be as a result of one or more of these elements. MOA theory on the other hand suggests that the availability of opportunities coupled with the individual’s level of motivation and ability provide a good indication of the individual’s propensity to engage in action (Syed Zwick, 2022). MOA elements and the supporting literature are presented in Table 2.

**Table 2:** Elements of MOA.

Motivation	Opportunity	Ability
1. Emotions	1. Opportunities	1 Abilities
2. Cognition	2. Timing	2. Skills
3. Decision making process	3. Resources	3. Knowledge
4. Desire	4. Location	
Su et al. (2020), Syed Zwick (2022)	Discua Cruz (2020), Fredericks (2020), Syed Zwick (2022)	Bandura (1977), Richards et al. (2019), Syed Zwick (2022)

Table 2 present elements that inform MOA namely; motivation, opportunity and ability. These elements are a precursor influencing EI (Fredericks, 2020). Hui-Chen et al. (2014, p. 728) are of the view that a combination of TPB together with MOA is likely to affect EI as indicated in Figure 1. Thus imply individuals’ intentions are likely to arise from attitude, subjective norms and perceived control behavior (PCB)/self-efficacy (Eid et al., 2019, p. 234). For example, attitude is regarded as a key stimulus in determining behavioral intentions (Jumani and Sukhabot, 2021, p. 18). With regard to the subjective norms, an entrepreneur’s behavioral traits, perception and motivation are modeled by external influences (Eid et al., 2019, p. 234). A case point in this regard would be when an individual desires to be entrepreneurial to emulate either a family member or business or is forced by circumstance. As such, external pressure can play a significant role in internalized commitments and perceived expected responses of others to an individual’s behavior (Randerson et al., 2020, p. 2). In terms of perceived control behavior (PCB) or self-efficacy, the process is driven by the individual’s capability (Hui-Chen et al., 2014) and judgment of his or her capabilities to organize and execute the course of action (Malebana, 2014).

### 3.2. Entrepreneurial action and the three stages

Despite many studies measured EA as a single construct (Bogatyreva et al., 2019), EA is not a single event but a dynamic and multiplicative process (Emami and Khajeheian, 2019, p. 1) that unfolds over time (Baum et al., 2007, p. 6). In the absence of such process, there would simply be no entrepreneurship (Swedberg, 2000, p. 26). For this study EA process inculcates all three stages associated with pursuing the entrepreneurial opportunity, that give rise to the formation of a new business venture or investment into something that will expand or improve business processes (Elia et al., 2020, p. 3). Table 3 presents the stages of EA as supported by literature.

**Table 3:** Elements of the stages of EA.

Entrepreneurial opportunity discovery (EODI)	Entrepreneurial opportunity evaluation (EOEV)	Entrepreneurial opportunity exploitation (EOEX)
1. Opportunity alertness	1. Rarity of the opportunity	1. Business opportunity
2. Opportunity formulation	2. Value	2. Developed a new market
3. Skills and knowledge	3. Limits on Competition	3. Entrepreneurial team
	4. Inimitability	4. Funding for a business opportunity
	5. Relatedness	
Meoli et al. (2020, p. 9), Su et al. (2020), p. 2, Calle et al. (2022)	Su et al. (2020), Teruel-Sánchez et al. (2021), Bosse et al. (2023, p. 3)	Dimov and Pistrui (2020, p. 2), Porter and Kramer (2019, p. 4), Maurer et al. (2022)

Table 3 presents the stages of EA (EODI, EOEV, EOEX) and the literature that supports them. This study regard EA process in the form of the stages leading to the exploitation of entrepreneurial opportunities (Wiklund et al., 2017b; Lerner et al., 2018, p. 3). The study adopted McMullen and Shepherd’s approach (McMullen and Shepherd, 2006, p. 133) by combining Discovery Theory (DT) and Creation Theory (CT) theories to conceptualize EA. This approach is also supported by Alvarez and Barney (2007, p. 17), who are of the view that certain actions are more likely to be effective in DT than CT or vice versa. DT and CT are suitable approaches to entrepreneurial opportunities, with regard to the formulation or discovery of opportunities, evaluation (McMullen and Shepherd, 2006, p. 133), and exploitation (Hills and Shrader, 1998, p. 54).

The first stage, EODI, refers to the intentional search for opportunities to be exploited for profit (Hsieh et al., 2007, p. 1,255). This is a vigorous and persistent action (Baron, 2007, p. 167; Esfandiar et al., 2019, p. 173) that entails the discovery or creation of entrepreneurial opportunities (Lins and Doktor, 2014, p. 22; Emami and Khajeheian, 2019, p. 1). This study adopted Kuckertz et al.’s (2017, p. 85) five key elements that suggest the EODI stage, namely: (i) opportunity alertness; such opportunities can arise as a result of exogenous opportunities that are created by shock in the market or system, or endogenous: informed by efforts, actions, reactions and enactment by entrepreneurs (Alvarez and Barney, 2007); (ii) search for markets; (iii) opportunity formulation in the sense that opportunities may be as a result of deliberate search or chance; (iv) skills and knowledge; this refers to the entrepreneur’s competencies to formulate or discover an entrepreneurial opportunity (Kuckertz et al., 2017, p. 85); (v) uncertainty: regular scanning of the environment for business opportunities that is informed by political, environmental, societal, technological, economical and legal (PESTEL) factors is key to the opportunity discovery stage (Rastkhiz et al., 2019, p. 67). Once the opportunity is discovered, the question is whether there is a desire and means to exploit it. Should the decision be not to continue with the opportunity any further, then that opportunity logically ceases to exist.

The second stage, EOEV, deals with the considerations in terms of desired feasibility and desired desirability of the opportunity (Keh et al., 2002, p. 126). The decision to act or not on the opportunity is a complex (Allinson et al., 2000, p. 31) and subjective exercise (Krueger, 1993, p. 6), owing to uncertainties (Obschonka and Stuetzer, 2017, p. 204). Drawing upon the creative theory, the EOEV stage is pivotal in terms of decisions leading up to acting on the opportunity or not (Allinson et al., 2000, p. 31).

This study adapted Haynie et al. (2009, p. 349) five key elements involved in the EOEV stage; (i) *Rarity* refers to uniqueness of the product or service in comparison to the substitute or what is already available in the system (Rastkhiz et al., 2019, pp. 191, 67); (ii) *Value*, in terms of this element, refers to the economic benefits of the opportunity in relation to cost (Shane and Venkataraman, 2000, p. 218); (iii) *Limits on Competition*, according to Haynie et al. (2009, p. 349), refers to how a business that controls valuable and scarce resources possesses competitive advantage; (iv) *Inimitability* serves as a barrier to entry by limiting entrance of possibly imitated products (Haynie et al., 2009, p. 349); (v) *Relatedness* refers to the extent to which the resources of the business can stretch to new markets (Haynie et al., 2009, p. 349). The next step after the evaluation is completed is the exploitation stage. This stage ensures that EA is realized (Ren et al., 2016, p. 468).

The third stage, EOEX, is the critical stage in terms of execution; without it, no goods, services or new business venture creation will be realized. This entails the gathering and recombining of required resources necessary to pursue opportunities that involve the creation of new ventures (Ren et al., 2016, p. 468). Informed by DT and CT theories, this study adopted Kuckertz et al.’s (2017) four key elements that suggest the EOEX stage. (i) *Business opportunity* refers to a deliberate action or decision that an entrepreneur has to take to translate entrepreneurial opportunity to meet human needs, consequently building wealth (Porter and Kramer, 2019, p. 4); (ii) *Develop new market* entails investigation of the market in terms of its needs, as well as the ability to discover opportunities and deploy required resources to meet the market requirements (Ardichvili et al., 2003, p. 105); (iii) *Entrepreneurial team* refers to the fact that entrepreneurial ventures, rather than being initiated by an individual entrepreneur, are most usually founded and driven by entrepreneurial teams (Lazar et al., 2020, p. 2); (iv) *Funding for business opportunity* refers to the fact that initial and working capital of entrepreneurial opportunity remains the most important step in launching any new venture or expanding an existing one (Kuckertz et al., 2017, p. 105). Such funding can be structured in many ways, either by the entrepreneur putting in his or her own funding, or through external funders or a combination thereof (Maurer et al., 2022, p. 808).

## 4. Hypothesis development: the relationship between EI and each of the stages of EA

As alluded by Meoli et al. (2020) that prior research between EI and EA found the correlation to be insignificant. Argue that the insignificant correlation between EI and EA could be because this relationship is not properly explained in entrepreneurship. Despite their assertion supporting the founding theory of intentions by Ajzen’s (1991) view that suggests intentions as proxy for action.

### 4.1. The relationship between EI and the EODI stage of EA

Opportunity discovery is a virtually instantaneous activity driven by intentions and capabilities (Hui-Chen et al., 2014, p. 727). EI and EODI are key activities required to initiate the entrepreneurial activity (Kautonen et al., 2015, pp. 4–5). This implies that in order for EODI to be realized, the entrepreneur needs to be deliberate and intentional about it, otherwise it may not be realizable (Galesic et al., 2016, p. 244; Calle et al., 2022, p. 2250015). Kautonen et al. (2015, p. 4) argue that entrepreneurial opportunities are not a matter of luck but are birthed by intentionality on the part of the entrepreneur (Hui-Chen et al., 2014, p. 727; Su et al., 2020, p. 2). This places EI and EODI in close proximity. Meoli et al. (2020, p. 9) are of the view that such proximity is reasonable and justifiable. We then posit that EI is likely to influence the identification of entrepreneurial opportunities leading to the entrepreneurial action taking place.

As such, the following hypothesis is stated:

*Hypothesis 1*: There is a positive relationship between entrepreneurial intention and the entrepreneurial opportunity discovery stage of EA.

Once the EODI stage is achieved, in most instances, the entrepreneur may want to know if such entrepreneurial opportunity is feasible and/or desirable to be pursued further or not. This leads to the next stage, which is EOEV.

### 4.2. The relationship between EI and the EOEV stage of EA

Key to the EOEV stage is the exercise to establish whether the opportunity can be exploited or not (Williams and Shepard, 2016, p. 366). This is a key process through which entrepreneurs make progress in decided whether to exploit their opportunities is by identifying, selecting, enrolling, and coordinating a network of stakeholders Bosse et al. (2023, p. 3). This exercise is loaded with decision on the side of the entrepreneur or his team in terms their perceived desirability and feasibility (Keh et al., 2002, p. 126). This denotes the extent to which an individual finds the prospect of pursuing the opportunity rewarding (Wiklund et al., 2017a, p. 13). The extent to which the opportunity is feasible is an exercise that depends on skills or past performance on a similar exercise (Wiklund et al., 2017a, p. 17). That provide the entrepreneur with the ability to perceive opportunities through taking advantage of existing resources (Teruel-Sánchez et al., 2021). However, the activities during this stage are not so obvious when coming to perceived desirability, which is subjective from person to person (Krueger, 1993, p. 6). This makes the exercise of whether the opportunity is desirable or not a bit cumbersome due to there being no standards or guidelines to follow (Keh et al., 2002, p. 126). However, Adam and Fayolle (2015, p. 36) argue that, irrespective of whether perceived feasibility or perceived desirability are established, intentionality will still be at play when coming to the evaluation exercise.

In some instances of establishing to exploit entrepreneurial opportunity the process may bypass the evaluation stage and move directly into exploitation stage. In those cases, the evaluation is more of a gut feeling than a calculated exercise, informed by perceived feasibility especially when dealing with novel opportunities (Keh et al., 2002, p. 126). In such cases a decision to pursue the opportunity or not is likely to be informed by the desired desirability. Su et al. (2020) argue that gut feeling cannot be ruled out in deciding on whether to pursue the opportunity or not. But even with the greatest of intentions, coupled with entrepreneurial opportunities, if the perceived desirability and to a certain extent the desired feasibility cannot be established, then such an opportunity can be thwarted (Esfandiar et al., 2019, p. 173; Suna et al., 2020, p. 2). Therefore the study purport that EI is likely to impact on the decision to evaluate the entrepreneurial opportunities leading to either the entrepreneurial opportunity is to be exploited or not.

Therefore, the following hypothesis is posited:

*Hypothesis 2*: There is a positive relationship between entrepreneurial intention and the entrepreneurial opportunity evaluation stage of EA.

### 4.3. The relationship between the EI and EOEX stages of EA

The birth of a business venture is associated with a successful discovery, evaluation and exploitation of an opportunity (Shane et al., 2003, p. 257). Once perceived feasibility and/or desirability is achieved, then the decision or action will be made on the entrepreneurial opportunity.

The EOEX stage is the apex of EA, entailing efforts by entrepreneur and his/her team combine resources together to a valuable product or service (Bosse et al., 2023, p. 3). Often these constructs get intertwined (Porter and Kramer, 2019, p. 4). The ontological assumption made with regard to the relationship between EI and EA is that EI has a positive correlation with EA. The extent of this correlation is the one that previous studies have found not to be significant (Adam and Fayolle, 2015, p. 45; Oliveira and Lima Rua, 2018, p. 508) and questionable (Meoli et al., 2020). However, it is a common understanding that entrepreneurial action starts as a result of something or a thought (Kautonen et al., 2015).

As such actual translation of intention into action is a key concept that its contribution to the entrepreneurial activity taking place cannot be underestimated (Esfandiar et al., 2019). Therefore the study purport that EI is likely to have a positive impact leading to the exploitation of entrepreneurial opportunities.

Therefore, the following Hypothesis is stated:

*Hypothesis 3*: There is a positive relationship between entrepreneurial intention and the entrepreneurial opportunity exploitation stage of EA.

**Figure 2:**
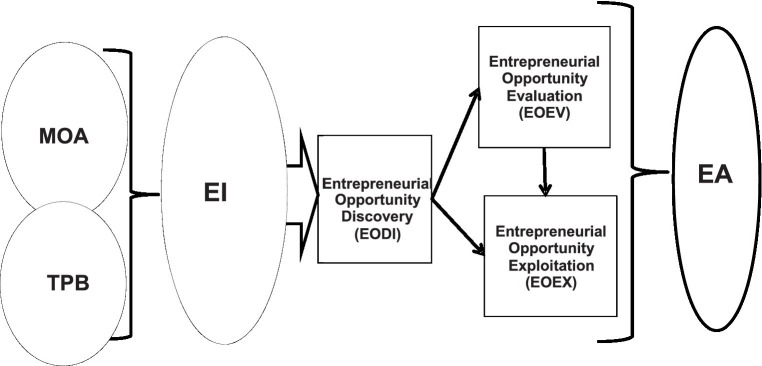
The theoretical model: Relationship between EI and the EA stages. *MOA, Motivation Opportunity Ability; TPB, Theory of Planned Behavior; EI, Entrepreneurial intention; EA, Entrepreneurial action; EODI, Entrepreneurial Opportunity Discovery (EODI); EOEV, Entrepreneurial Opportunity Evaluation; EOEX, Entrepreneurial Opportunity Exploitation; PCB, Perceived Control Behavior. Own compilation.

In order to provide insights into how EI translates into decisions for new venture creation, Figure 2 depicts the theoretical model illustrating the relationship between EI and the stages of EA that Esfandiar et al. (2019, p. 173) suggest is key to stimulate action.

Figure 2 depicts the relationship between EI and the stages EA. EI relates to pre-venture activities that influence the entrepreneur’s state of mind in directing his or her attention toward action (Esfandiar et al., 2019, p. 173). On the other hand, EA consists of vigorous and persistent activities that are likely to lead to an entrepreneurial event (Swedberg, 2000, p. 26): discovery, evaluation and exploitation (Baron, 2007, p. 167).

**Figure 3:**
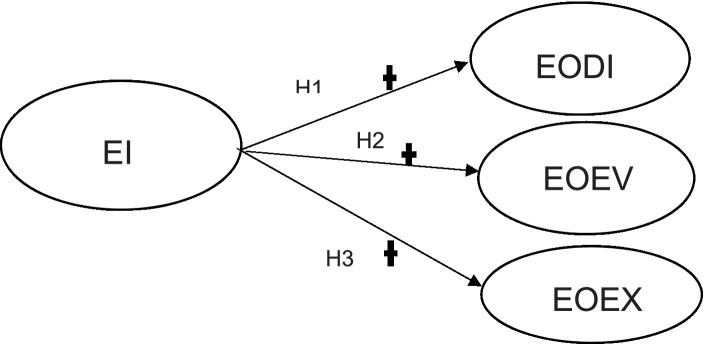
The hypothesized model: relationship between EI and the EA stages. Own compilation.

Furthermore, the theoretical model is used to illustrate the hypothesized model in Figure 3 depicting the relationship between EI and the stages.

Figure 3 indicates how the hypotheses will be measured and the likelihood of EI (independent variable) having a positive effect on all three stages of EA (dependent variables) as per the hypotheses of the study.

## 5. Methods

**Figure 4:**
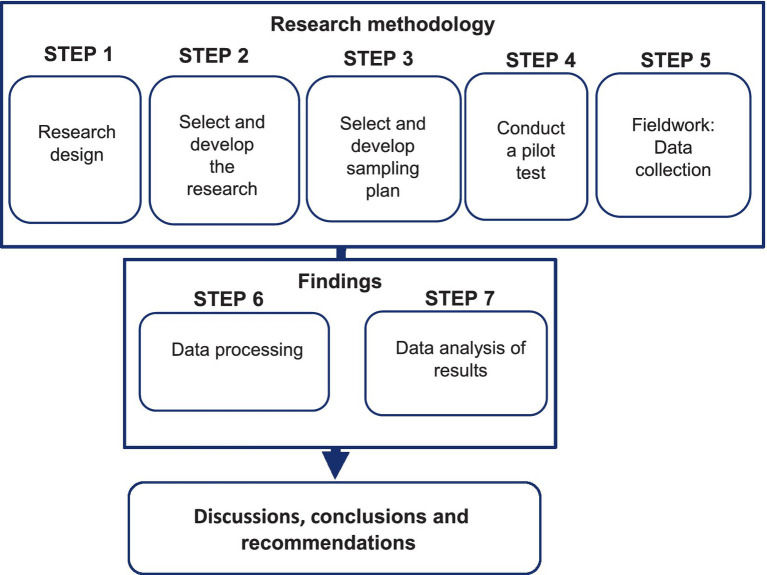
The research design: own compilation.

An eight-step process was followed to indicate how the methodology was carried out in the study and is presented in Figure 4.

Figure 4 illustrates the methodology followed for this study, whereby the process outlined the research design for this study that is underpinned by literature that supports this study. The primary data-collection techniques related to the survey method are provided and discussed: questionnaire, sample, sampling technique used, why a chosen sample, and questionnaire administration. The research hypotheses are outlined and a justification for each hypothesis adopted. The measurement for reliability and validity of this study was also discussed, in terms of SEM technique or procedures engaged to analyze the data; through goodness-of-fit indices, CFA and EFA. The last step consists of discussions, conclusion and recommendation for future research.

### 5.1. Sampling procedure

The study followed a quantitative approach to investigating 597 South African entrepreneurs. An entrepreneur in this case refers to an owner-manager of a business (Botha and Pietersen, 2020, p. 529). In the sample, the status of the business was indicated by the age of the business with the option of almost started (41.7%) and those who already had existing businesses (58.3%). Businesses that were “almost started” refers to the nascent phase, which consists of entrepreneurial individuals who were about to start their business ventures or those who had started and operated their businesses for less than a year. The “existing businesses” refers to ventures that have been in existence for longer than 1 year and onwards (Hartanto et al., 2017, p. 1131). Data was collected through structured self-administered questionnaires. The questionnaire was administered to a database of 1,000 entrepreneurs which was obtained from Small Enterprise Development Agency (SEDA). From this database, 597 respondents completed the survey and therefore the response rate was 59.7%. Nascent entrepreneurs 249 (41.7%) and Existing entrepreneurs 348 (58.3%). The survey was conducted with 30 entrepreneurs *via* email or hard copies. The results from the pilot study, in terms of the face validity, revealed that the instrument was generally understood and there were no changes required.

## 6. Measures

### 6.1. Entrepreneurial intention survey development

EI is a well-researched construct, making it a popular construct in entrepreneurship literature (Van Gelderen et al., 2015; Meoli et al., 2020, p. 2). As the result of this popularity, there are numerous instruments in literature to measure it, such as the one by Guerrero et al. (2009, p. 8) that is adopted for this study. As much as the two groups were tested (nascent and existing entrepreneur), a preliminary analysis was conducted to establish whether the study would result in different CFA models to be considered due to metric invariance. Metric invariance refers to a statistical property of measurement that indicates that the same construct can be measured across specified groups (Ho, 2020). As such metric invariances were established, this confirmed that metric invariance could be used for this study (Ho, 2020). A five-point Likert-type response scale was used to this effect (with 1 indicating “strongly disagree” and 5 “strongly agree”) and responses were scored as casual indicators of intention, using the process approach to modeling within an SEM framework. This included items such as, “I am ready to do anything to be an entrepreneur … I will make every effort to start and run my own business” (refer to all items that inform EI in the Appendix). The scale used presented a KMO = 0.913, *χ*^2^ = 657.68 and coefficient α = 0.924. It is of the utmost importance that the measurement instrument to be valid and reliable in order to be considered trustworthy (Sekaran and Bougie, 2013, p. 225). Therefore Guerrero et al.’s (2009) scale met the validity and reliability threshold as it was considered reliable and valid; hence it was adopted.

### 6.2. Stages of entrepreneurial action survey development

As indicated that the stages of EA consist of EODI, EOEV and EOEX (Shane and Venkataraman, 2000, p. 218; McMullen and Shepherd, 2006, p. 134; McMullen et al., 2007, p. 273). These entail actions that surround the pursuing of the entrepreneurial opportunity, from the idea being formulated right up to giving rise to the formation of a business (Shane et al., 2003, p. 257). This study adopted the scale by Kuckertz et al. (2017, p. 84), to measure EODI and EOEX stages. This is owing to how their scale measures its content domains, and items that inform EODI and EOEX stages of EA. The original scale responses were scored on a Likert scale from 1 to 7, and this study adapted it from 1 (strongly disagree) to 5 (strongly agree) in order to align the scale with the rest of the instrument used. The scale included elements for EODI such as: “I am always alert to business opportunities … I research potential markets to identify business opportunities,” and for EOEX “I have set up an organization to pursue a business opportunity I perceived, based on a business opportunity … I have developed a new market” (refer to all items that inform stages of EA in the Appendix). The reliability and validity of the measurement were tested and established by employing exploratory and confirmatory factor analyses alongside other correlational analyses (Kuckertz et al., 2017, p. 84). As such the coefficients α for EODI scale = 0.87, and EOEX = 0.79, were found reliable and valid for the scale to be considered for this study. In terms of evaluating the entrepreneurial opportunities, this study adapted the (Haynie et al.’s 2009, p. 349) measuring scale to determine EOEV. The scale consisted of five items that determined EOEV; for example, Value coefficient α = 3.02, Rarity coefficient α = 1.29, Inimitability coefficient α = 0.28 Limits to competition, coefficient α = 1.78 and Relatedness coefficient α = 2.27. Other than with the limits of competition, all items were above the accepted threshold for the coefficient alpha being ≥0.70 for the established instrument.

## 7. Results

### 7.1. Demographics of the sample

The net total of 597 responses were captured after the discarding of incomplete data; 49.6% were male respondents while 47.1% were female respondents. The respondents were well represented in all of the provinces in South Africa except Limpopo, which had no respondents. The results indicated that three provinces accounted for 87.1% of the results of the data collected, Gauteng having the majority at 51.6%, followed by North West, 19.4%, then Eastern Cape, 16.1%; the balance of 12.9% of the respondents was received from five other provinces. Regarding the respondents’ age, the majority (60.4%) of respondents were between 25 and 44 years old. The status of the business was indicated by the age of the business with the option of almost started (41.7%), relating to nascent entrepreneurs, and those who already had existing businesses (58.3%). The sample was collected randomly across all races.

### 7.2. Validity and reliability of the measurement instrument

The validity and reliability of the measurement instrument was established and confirmed (Sekaran and Bougie, 2013, p. 225) prior to measuring and presenting the inferential statistics of the constructs. Reliability and validity tests conducted demonstrated the rigor and the trustworthiness of study findings (Roberts and Helena, 2006, p. 41). Exploratory factor analysis (EFA) was conducted on the EI and EA scales, since the measuring instruments used for these scales were adapted. EFA can be employed in the evaluation of theories and the validation of measurement instruments, such that factors or latent factors can be identified to parsimoniously explain the covariation (Watkins, 2018, p. 219).

Thus the EFA was employed since there were changes in wording. For example in the original instrument pertaining to EI and the stages of EA, participants were required to answer the questionnaire in the order of importance in line with the five Likert scales. In the adapted version employed in this study, participants were required to answer by choosing 5 for “strongly agree”, 4 “agree”, 3 “neutral”, 2 “disagree”, and 1 “strongly disagree” with the statements.

As indicated, the study had two entrepreneurially distinct groups, namely nascent entrepreneurs and existing entrepreneurs. As such, metric invariance was considered and confirmed that metric invariance could be used (Ho, 2020). In this regard the nested model was employed, assuming the unconstrained model to be correct. It indicated a value of *p* of 0.157 for the structural weights, therefore indicating no statistical significance, as the value was above 0.05. Thus, metric invariance between the two groups was accepted. Structural covariances also showed no statistical significance and only measurement residuals showed statistical significance. Thus metric invariance can be assumed for the analysis, as indicated by Ho (2020). Ho stated that if subsequent analyses use the measure as a latent variable, differences in measurement residual variances will not impact on inferences about group differences in prediction, as long as the loading is equal across groups. Therefore the two sub-samples of nascent and established entrepreneurs could be combined to form one group to conduct the further analyses on. Table 4 indicates the results.

**Table 4:** The metric invariance.

Model	DF	CMIN	*p*
Measurement weights	41	50.063	0.157
Structural covariances	51	58.09	0.216
Measurement residuals	94	151.349	0.000^a^

a indicated statistical significance (*p* < 0.001).

As indicated, the measurement instruments were adapted from reputable and well-cited authors. However, the Cronbach-alpha coefficients of composite reliability and discriminant validity for this study were also conducted to confirm the validity and reliability of these measurement instruments.

### 7.3. Exploratory factor analysis

The rule of thumb in terms of the suitability of data is that the bigger the sample size the better the factorization of data (Pallant, 2011, p. 18). The sample size for this study stood at 597 respondents, therefore this number was deemed good enough to perform factor analysis. Other statistical tests such as the Kaiser-Meyer-Olkin measurement (KMO) that must exceed the minimum value of 0.6 and the Bartlett’s test of sphericity (*p* < 0.001) were utilized to test the factorability of the correlation matrix.

The results of the EFA on EI and later those of the stages of EA are presented in the following sections. The test followed employing EFA firstly commences with the Kaiser-Meyer-Olkin (KMO) test for sampling adequacy and Bartlett’s test of sphericity, which assess the suitability of the data for factor analysis. Bartlett’s test of sphericity threshold should be significant (*p* < 0.05) for the factor analysis to be considered appropriate (Kline, 2014). The KMO index ranges from 0 to 1 and a minimum value of 0.6 is considered appropriate for factor analysis.

#### 7.3.1. Exploratory factor analysis: EI

In terms of EFA for EI, the KMO value for EI is 0.876, exceeding the minimum value of 0.6. The Bartlett’s test of sphericity showed statistical significance (p < 0.001), therefore supporting the factorability of the correlation matrix. The results identified EI as a uni-dimensional construct, based on the eigenvalue exceeding 1 criterion. The eigenvalue for the EI factor was 3.642 and explained 72.84% of the total variance. The final factor loadings are presented below in Table 5.

**Table 5:** Factor loadings from the EFA for the factor representing EI.

Items	Factor 1
e4 B5	0.703
e7 B6	0.875
e8 B7	0.816
e9 B8	0.840
e11 B9	0.827

The internal consistency (reliability) for the EI factor was measured using Cronbach Alpha and the value is 0.902, which is above the general threshold of 0.7 (Hair et al., 2014) for instruments from previous research. In the following section, EFA was conducted for each of the EA stages separately.

#### 7.3.2. Exploratory factor analysis: EODI stage of EA

The KMO value for EODI was 0.898, exceeding the minimum value of 0.6. The Bartlett’s test of sphericity showed statistical significance (*p* < 0.001), therefore supporting the factorability of the correlation matrix. The results identified EODI as a uni-dimensional construct based on the eigenvalue exceeding 1 criterion. The eigenvalue for the EODI factor was 3.776, which explained 75.52% of the total variance. The final factor loadings are presented below in Table 6.

**Table 6:** Factor loadings from the EFA for the factors representing EODI.

Items	Factor 1
e26 B10	0.776
e27 B11	0.866
e28 B12	0.872
e29 B13	0.835
e30 B14	0.815

The internal consistency (reliability) for EODI was measured using Cronbach Alpha and the values were 0.919. The reliability for the EODI factor was above the generally accepted threshold of 0.7 (Hair et al., 2014).

#### 7.3.3. Exploratory factor analysis: EOEV stage of EA

The KMO value for EOEV was 0.898, exceeding the minimum value of 0.6. The Bartlett’s test of sphericity showed statistical significance (p < 0.001), therefore supporting the factorability of the correlation matrix. The results identified EOEV as a uni-dimensional construct based on the eigenvalue exceeding 1 criterion. The eigenvalue for the EOEV factor, which is 2.939, explained 58.78% of the total variance. The final factor loadings are presented below in Table 7.

**Table 7:** Factor loadings from the EFA for the factors representing EOEV.

Items	Factor 1
E36 B15	0.648
E37 B16	0.697
E38 B17	0.826
B18	0.683
E40 B19	0.624

The internal consistency (reliability) for EOEV was measured using Cronbach Alpha and the value was 0.819. The reliability for EOEV factor was above the general threshold of 0.7 (Hair et al., 2014).

#### 7.3.4. Exploratory factor analysis: EOEX stage of EA

The KMO value for EOEX was 0.825, exceeding the minimum value of 0.6. The Bartlett’s test of sphericity showed statistical significance (p < 0.001), therefore supporting the factorability of the correlation matrix. The results identified EOEX as a uni-dimensional construct based on the eigenvalue exceeding 1 criterion. The eigenvalue for factor 1, which is 2.955, explained 73.87% of the total variance. The final factor loadings are presented below in Table 8.

**Table 8:** Factor loadings from the EFA for the factors representing EOEX.

Items	Factor 1
E41 B20	0.787
E42 B21.	0.833
E43 B22	0.841
E44 B23	0.768

The internal consistency (reliability) for EOEX was measured using Cronbach Alpha and the value was 0.880. The reliability for the EOEX factor was above the general threshold of 0.7 (Hair et al., 2014).

### 7.4. CFA and structural equation modeling

**Figure 5:**
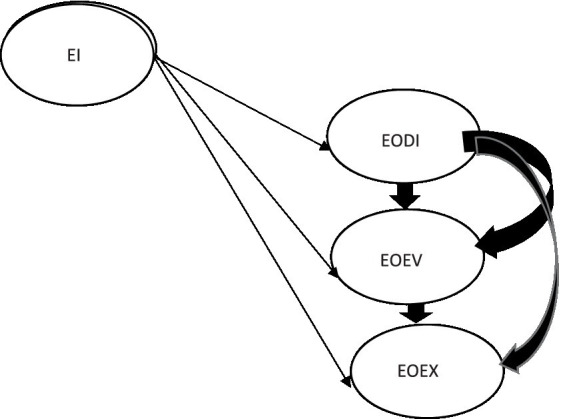
SEM model in relation to EI and the stages of EA.

For the model to be considered appropriate there are a number of goodness-to-fit indices that must be met (Hair et al., 2014). The goodness-of-fit test was performed to ascertain the model of fit as presented in Table 6. Therefore the CFA was employed and confirmed model fit for the relationship between EI and the stages of EA. SEM tested the relationship between EI and the stages of EA as indicated in Figure 5.

Figure 5 presents the variables of the study; depicting the relationship EI construct and the three stages of EA. The model was tested for consistency with the observed data by means of a set of generally accepted fit indices in order to establish whether the current data fits the relationship between EI and the stages of EA by following the process approach. A model with the following goodness-of-fit indices indicates acceptable fit: RMSEA values between 0.05 and 0.08; CFI, IFI and TLI above 0.9; and the CMIN/df value smaller than 3 (Hair et al., 2014, p. 579).

Table 9 below reveals the goodness-of-fit indices of the structural model representing the relationship between EI and the stages of EA.

**Table 9:** Goodness-of-fit indices for the structural model for the relationship between EI and the stages of EA.

Model	CMIN ($x^{2}$)	df	*p*	CMIN/df	RMSEA	CFI	TLI	IFI	SRMR
Model 1	542.643	129	0.000^a^	4.207	0.073	0.944	0.934	0.945	0.0457
Acceptable levels	–	–	–	< 3 or <5	≤ 0.08	≥ 0.90	≥ 0.90	≥ 0.90	< 0.08

a Indicated statistical significance (*p* < 0.001).

The model presented in Table 6 indicated that the CMIN/df (4.207), RMSEA (0.073) and SRMR (0.0457) were below the recommended thresholds and thus indicated acceptable fit. The CFI (0.944), TLI (0.934) and IFI (0.945) were above the 0.90 acceptable level, thus confirming an acceptable model fit.

### 7.5. Convergent validity

The composite reliability values for each of the constructs of the EI and the stages of EA were tested and results as presented in Table 10 below.

**Table 10:** Convergent validity.

	Entrepreneurial intension	Entrepreneurial opportunity discovery	Entrepreneurial opportunity evaluation	Entrepreneurial opportunity exploitation
CR	0.871	0.919	0.825	0.882

The results indicated that EI; EODI, EOEV and EOEX have a composite reliability value that is above the 0.70 threshold (Anderson et al., 2010). Thus suggest that convergent validity is achieved.

### 7.6. The regression effect

Table 11 the regression effect of the relationship between EI and the stages of EA.

**Table 11:** Factor loadings representing EI and the stages of EA.

Relationships	Regression weights	Standardized regression	Label
EI–EODI	0.802	0.731	***
EI–EOEV	0.738	0.664	***
EI–EOEX	0.644	0.493	***

***Statistical relationship.

**Figure 6:**
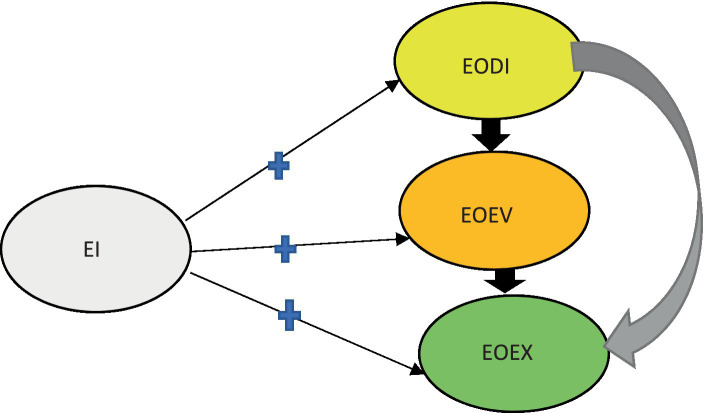
Process approach; EI effect on the stages of EA.

Table 11 represents the relationship between EI and the stages of EA. The study found a correlation between EI and the stages of EA, as depicted by Figure 6.

Figure 6 indicate the process in the relationship between EI and the stages of EA. The study found that EODI, EOEV and EOEX stages had a positive, statistically significant relationships with EI. The strength of this relationship was strong (larger than 0.5) for the EODI and EOEV, and moderate (between 0.3 and 0.5) for EOEX. This suggests that EI has a direct effect on each of the three stages of EA.

## 8. Discussion

The final step in the research process is the presentation of the findings in the light of the stated hypotheses by drawing conclusions and recommendations which are supported by the existing literature. The assessment of measurement model’s reliability and validity was conducted through the application of CFA procedures. The findings of the study suggest that the model had an acceptable construct validity and reliability. All the measurement scales revealed convergent validity, indicating that each item had statistically significant loadings on each factor specified.

The study tested the effect of the relationship between EI and each of the stages of EA by applying a process approach from the premise of theories that suggest the impact of intention on action (Ajzen, 1991). Based on the SEM analyses conducted, the results indicated that the model fit. The indices included CFI = 0.944, TLI = 0.934 and TLI = 0.945, values that were all above the 0.9 threshold, RMSEA = 0.073 and SRMR = 0.0457 which were below the threshold of 0.08. Thus posits EI had a positive, statistically significant relationship with all three of the stages of EA (EODI, EOEV and EOEX). This implied that all three hypotheses are positive in relation to the effect of EI on the stages of EA. As such the effect of EI impacting on each of the stages of EA was established as summarized in Table 12.

**Table 12:** Hypotheses tested.

		Regression weights	Standardized regression	Label		The strength of the relationship
H1	There is a positive relationship between EI and EODI stage of EA	0.802	0.731	***	Positive	> 0.5
H2	There is a positive relationship between EI and EOEV stage of EA	0.738	0.664	***	Positive	> 0.5
H3	There is a positive relationship between EI and EOEX stage of EA	0.644	0.493	***	Positive	0.3 ≥ ≤ 0.5

***Statistical relationship.

Table 12 summarizes the findings of the study (Annexure 1). The study employed a model that integrated elements of the Theory of Planned Behavior (TPB) together with Motivation Opportunity Ability (MOA) theory to form elements of EI and DT and CT theories, to suggest the stages of EA as depicted in Figure 2. The study found a positive correlation between EI and the stages of EA. This suggest individual’s level of intentions coupled with entrepreneurial opportunities is a good predictors to engage into the entrepreneurial action (Syed Zwick, 2022). The study followed a process approach to investigate the relationship between EI and the three stages of EA and found a strong positive relationship between EI and EODI and EOEX and moderate with EOEX.

The study found a strong positive relationship between EI and EODI. This is supported by literature that find such relationship between EI and EODI as intertwined and fundamental in the process leading to EA taking place (Liu et al., 2022). In the sense that entrepreneurs find that opportunity alertness and risk-taking propensity have positive effect to their intentions (Calle et al., 2022). In the sense that in order for the opportunity to be discovered this will be initiated by EI process (Bapoo et al., 2022) or deliberate action stemming from intentions (Karimi et al., 2016, p. 187; Esfandiar et al., 2019, p. 173). Therefore, the study found a positive relationship between the EI and the EODI stage. The strength of this relationship was strong (larger than 0.5) for the EODI. Thus suggest hypothesis H1a is supported as per the literature and empirical results presented.

Once EODI stage is realized, the second stage that ascertains if the opportunity makes sense to the entrepreneur to pursue further (McMullen and Shepherd, 2006). In terms of the second stage the relationship between EI and the EOEV stage, the study found this relationship positive. Whereby the strength of this relationship was strong (larger than 0.5) for EOEV. This suggest EOEV process are driven by great sense of intentionality (Haynie et al., 2009, p. 349). That entail activities such as identifying, selecting, enrolling and coordinating a network of stakeholders in pursuance of the entrepreneurial opportunity (Bosse et al., 2023, p. 3). The study found EOEV to be a crucial exercise that requires intentionalilty, skills and attitude leading to a decision or action to take advantage of existing opportunities (Teruel-Sánchez et al., 2021). Therefore hypothesis H2 is supported as per the literature and empirical results presented.

The last phase is the relationship between EI and the EOEX. This stage is key for the entrepreneurial event to be realizable (Porter and Kramer, 2019, p. 4; Maurer et al., 2022, p. 808). The results of the study found a positive relationship between the EI and the EOEX stage. The strength of the relationship somewhat moderate (between 0.3 and 0.5) for EOEX. Much as the literature support the link between EI and EOEX however not all EI lead to EOEX taking place. This is supported by literature, that once an entrepreneurial opportunity is discovered and evaluated, then the entrepreneur must decide whether to abort or exploit it (Shane and Venkataraman, 2000). However, we found that the entrepreneur’s intentions are key to making the exploitation stage realizable (Alvarez, 2005, p. 13). This suggests that the entrepreneur’s intention to exploit the entrepreneurial opportunity is pivotal at this juncture (Dimov and Pistrui, 2020, p. 2) and that EI is not only necessary during the EODI and EOEV stages but also during the EOEX stage. Therefore hypothesis H3 is also supported as per the literature and empirical results presented.

## 9. Conclusion

### 9.1. Contribution to theory and practice

As entrepreneurship research continues to be studied from different perspectives in order to broaden the knowledge and application from a theoretical point of view (Teruel-Sánchez et al., 2021). The study of the relationship between EI and EA remain relevant in entrepreneurship research due to its contribution leading to the entrepreneurial activity taking place (Esfandiar et al., 2019). However as stated that there still discourse in entrepreneurship literature on how this relationship is measured (Meoli et al., 2020). Most studies measure this relationship from a binary point of view (Bogatyreva et al., 2019, p. 309), even though EA is can be argued to be multiplicative process (Emami and Khajeheian, 2019, p. 1) that unfolds over time (Baum et al., 2007, p. 6).

This study has made main contributions to the literature through the development and testing of a model that applies a process approach. Thus imply the relationship between EI, EODI, EOEV and EOEX is measured in terms of its logical flow (Elia et al., 2020, p. 3). By following this approach broadens our understanding of what pertains to EA. As action is not only limited to when the product or the opportunity is exploited but also extends to other entrepreneurial activities that are. By following a process approach aid in breaking down entrepreneurial activities (Fini and Grimaldi, 2017). From EI right up to when EA is actualized (Khan et al., 2022).

To arrive at best possible EA levels suggest that scholars should go beyond traditional binary approach and explore other possible processes that are likely to extract outcomes that are limited to a binary measurement (Paoloni et al., 2020). In doing so requires that parameters should be clearly defined and measurable (Cooper and Schindler, 2011, p. 142). As EA process occurs over time (Emami and Khajeheian, 2019, p. 1). Therefore to illustrate the impact of EI to the stages of EA, the study employed the process approach. Through incorporating TPB in conjunction with MOA theory to formulate EI (independent variable) and the Discovery Theory and the Creation Theory to formulate the stages of EA (dependent variables). In so doing the study contributed to theory by aligning these four theories in support of a process to measure the relationship between these. In so doing the study established the statistical significant correlation between EI and all three stages of EA. This suggest the positive significant relationship that is likely to have a positive impact in minimizing EI-EA gap in support of Meoli et al.’s (2020) assertion that EA levels could be higher than what they are currently recorded.

The period between EI and ultimately the opportunity is exploited is often engulfed by uncertainties that may result either in the opportunity fully exploited or abandoned half way (Marinacci, 2015, p. 1023). The process between the discovery stage and when the opportunity is fully exploited suggest that there may be some entrepreneurial activities that took place (Wiklund et al., 2017b). Even if such activities did not end up in the opportunity being fully exploited. As long as such activities can be established, therefore the relationship between EI and EODI, EOEV and EOEX is likely to be established (Su et al., 2020).

Therefore, this study contributes to practice, by adding its support to studies that suggest that in order for EA to be realized that EI should be in place to initiate the process leading to stages of EA to take place. Furthermore, the understanding and application of measuring the relationship between EI and the stages of EA correctly, the study provided a useful guideline to which education and training programs can adopt to measure EA levels more correctly. Supporting Meoli et al.’s (2020) assertion that the insignificant correlation between EI and EA could have been as a result of this relationship not properly measured in entrepreneurship.

### 9.2. Limitations and future direction

There are limitations that should be noted in considering the results of this study. First, as much as the scales used in this study were from a valid instrument, some of the elements, in particular from the EI scale, were deemed ambiguous – for example items such as “I have a strong intension of ever starting a business; I am ready to do anything to be an entrepreneur.” As most respondents are already in established businesses, as such this line of questioning is likely to create errors in responding. Future research should create scales relevant to those already in established businesses to test their recurring EI or instead of investigating their entrepreneurial intents to focus should shift to their entrepreneurial mindset. This would be a better proposition to individuals who are already entrepreneurs because of their ability to sense, and act on entrepreneurial opportunities. As entrepreneurship scholars in general like to engage in cognitive research that seeks to understand how individuals identify entrepreneurial opportunities and act upon them (McMullen and Shepherd, 2006, p. 132). Second, as the EA process happens over a period of time, future investigations would benefit immensely from longitudinal and experimental research, as opposed to a cross-sectional study. Lastly, even though this study offers certain limitations associated with geographical area. Therefore, a comparative study could be developed in other geographical areas to compare results in order to avoid bias as a result of socioeconomic conditions.

## Data availability statement

The raw data supporting the conclusions of this article will be made available by the authors, without undue reservation.

## Author contributions

All authors listed have made a substantial, direct, and intellectual contribution to the work and approved it for publication.

## Conflict of interest

The authors declare that the research was conducted in the absence of any commercial or financial relationships that could be construed as a potential conflict of interest.
